# A Novel Actin Binding Drug with *In Vivo* Efficacy

**DOI:** 10.1128/AAC.01585-18

**Published:** 2018-12-21

**Authors:** Akshaya Ravichandran, Mengxin Geng, Kenneth G. Hull, Jing Li, Daniel Romo, Shi-En Lu, Aaron Albee, Christopher Nutter, Donna M. Gordon, Mahmoud A. Ghannoum, Steve W. Lockless, Leif Smith

**Affiliations:** a Department of Biology, Texas A&M University, College Station, Texas, USA; b Department of Chemistry and Biochemistry, CPRIT Synthesis and Drug-Lead Discovery Laboratory, Baylor University, Waco, Texas, USA; c Department of Chemistry, Natural Products LINCHPIN lab, Texas A&M University, College Station, Texas, USA; d Department of Biochemistry, Molecular Biology, Entomology and Plant Pathology, Mississippi State University, Starkville, Mississippi, USA; e Department of Biological Sciences, Mississippi State University, Mississippi State, Mississippi, USA; f Center for Medical Mycology, University Hospitals of Cleveland/Case Western Reserve University, Cleveland, Ohio, USA

**Keywords:** actin binding proteins, anticancer therapy, antifungal therapy, candidiasis, occidiofungin

## Abstract

Occidiofungin is produced by the soil bacterium Burkolderia contaminans MS14 and is structurally similar or identical to the burkholdines, xylocandins, and cepacidines. This study identified the primary cellular target of occidiofungin, which was determined to be actin. The modification of occidiofungin with a functional alkyne group enabled affinity purification assays and localization studies in yeast. Occidiofungin has a subtle effect on actin dynamics that triggers apoptotic cell death. We demonstrate the highly specific localization of occidiofungin to cellular regions rich in actin in yeast and the binding of occidiofungin to purified actin *in vitro*. Furthermore, a disruption of actin-mediated cellular processes, such as endocytosis, nuclear segregation, and hyphal formation, was observed. All of these processes require the formation of stable actin cables, which are disrupted following the addition of a subinhibitory concentration of occidiofungin. We were also able to demonstrate the effectiveness of occidiofungin in treating a vulvovaginal yeast infection in a murine model. The results of this study are important for the development of an efficacious novel class of actin binding drugs that may fill the existing gap in treatment options for fungal infections or different types of cancer.

## Abstract (precis)

Occidiofungin is produced by the soil bacterium Burkolderia contaminans MS14 and is structurally similar or identical to the burkholdines, xylocandins, and cepacidines. This study identified the primary cellular target of occidiofungin, which was determined to be actin.

## INTRODUCTION

Fungal infections caused by pathogens that are resistant to commonly used classes of antifungals are becoming increasingly prevalent (1–4). The rise in candidemia caused by non-*albicans Candida* spp. and the increase in azole resistance (5–9) are alarming and support the need for new antifungals. This problem is expected to be exacerbated by the presence of the multidrug-resistant fungus Candida auris in hospitals (7). An example of a fungal infection that is rapidly developing resistance to currently available forms of treatment is vulvovaginal candidiasis (VVC) (10). VVC will affect approximately 75% of all women, and 5% to 10% of all women will develop recurrent VVC (RVVC) (11–13). There have been no new therapeutic developments for decades for recurrent VVC.

Clinically approved antifungals primarily comprise members of the polyene, echinocandin, and azole family of compounds. Azoles and polyenes primarily target ergosterol production and bind to ergosterol, respectively, disrupting the fungal membrane. The echinocandins are synthetically modified lipopeptides that originate from a natural cyclic peptide compound produced by fungi. This group selectively inhibits 1,3-β-glucan synthesis by functioning as a noncompetitive inhibitor of 1,3-β-glucan synthase (14–17). The prevalence of echinocandin- and azole-resistant fungal pathogens and the limited spectrum of activities of those compounds are major issues contributing to the need for a new class of antifungals. Additionally, current antifungal treatments lead to abnormal liver and kidney function tests and have limitations with respect to their spectrum of activities and toxicities (18, 19). These limitations and toxicity problems have created an urgent need to identify antifungal compounds that have a novel mechanism of action (20).

**FIG 1:**
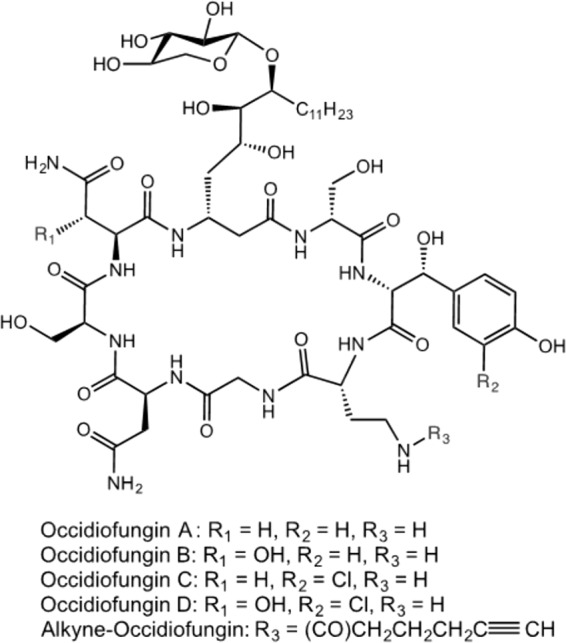
Covalent structure of occidiofungin A-D and alkyne-OF.

Occidiofungin is a nonribosomally synthesized glycolipopeptide produced by the soil bacterium Burkholderia contaminans MS14 (21). It is a cyclic peptide with a base mass of 1,200 Da (Fig. 1) (21). Occidiofungin has a wide spectrum of activities against filamentous and nonfilamentous fungi and minimal toxicity in an animal system (21, 22). We previously demonstrated that the mechanism of action of occidiofungin differs from the primary modes of action of the three common classes of antifungals (23, 24). Additional assays indicated that occidiofungin rapidly induces apoptosis in yeast cells at the MIC (24). Interestingly, a critical threshold concentration of occidiofungin is required for its observed fungicidal activity. Occidiofungin has little to no impact on the growth rate of yeast at subinhibitory concentrations (23). Preliminary toxicological analyses of occidiofungin using a murine model indicated that it was well tolerated at concentrations of 10 to 20 mg/kg (22). Intravenous administration of occidiofungin to mice at a dose of 5 mg/kg was carried out with minimal induced toxicity. Blood chemistry analyses and histopathology performed on multiple organs showed a transient nonspecific stress response with no damage to organ tissues (25). Altogether, the data suggest that occidiofungin is a promising candidate for development as a clinically useful antifungal agent. This report describes studies to identify the molecular target of occidiofungin and determine its efficacy in a murine model of vulvovaginal candidiasis.

## RESULTS

### Spectrum of activities of occidiofungin against clinically relevant fungi.

Because of its unique mechanism of action, occidiofungin has submicromolar activity against azole- and echinocandin-resistant strains of fungi. Strains of Candida albicans, Candida glabrata, and Candida parapsilosis that were resistant to fluconazole and caspofungin were sensitive to occidiofungin (see Table S1 in the supplemental material). Furthermore, strains of C. auris were sensitive to occidiofungin at submicromolar concentrations. Strains of Candida parapsilosis and C. neoformans that were resistant to treatment with caspofungin were found to be susceptible to treatment with occidiofungin. Occidiofungin was also found to be active against *Aspergillus*, *Mucor*, *Fusarium*, *Rhizopus*, and azole- and terbinafine-resistant strains of the dermatophyte *Trichophyton*. A summary of the results, reported in Table S1, indicates that occidiofungin has activity against filamentous and nonfilamentous fungi at submicromolar concentrations and has a broader spectrum of activities than those of other clinically available antifungals.

### Alkyne derivatization of occidiofungin.

Occidiofungin was chemically modified with a terminal alkyne through acylation of the free amino group of the diaminobutyric acid residue at position 5 (see Fig. S1 for subsequent click chemistry, Sharpless-Hüisgen cycloaddition). The analog enabled fluorescence microscopy and binding studies. The modified occidiofungin, alkyne-OF, had an 8-fold reduction in activity with MICs of 1 and 0.5 µg/ml against Saccharomyces cerevisiae BY4741 and Schizosaccharomyces pombe 972h-, respectively (see Table S2). Double-stranded DNA breaks, the generation of reactive oxygen species (ROS), and the externalization of phosphatidylserine were observed in the alkyne-OF-treated cells, supporting the same mechanism of action as native occidiofungin (see Fig. S2A to C). Although this alkyne modification moderately reduced the inhibitory activity of the compound, the functionalized derivative has the same apoptotic bioactivity and was therefore used to identify the fungal target.

### *In vivo* analysis of the intracellular localization of occidiofungin.

**FIG 2:**
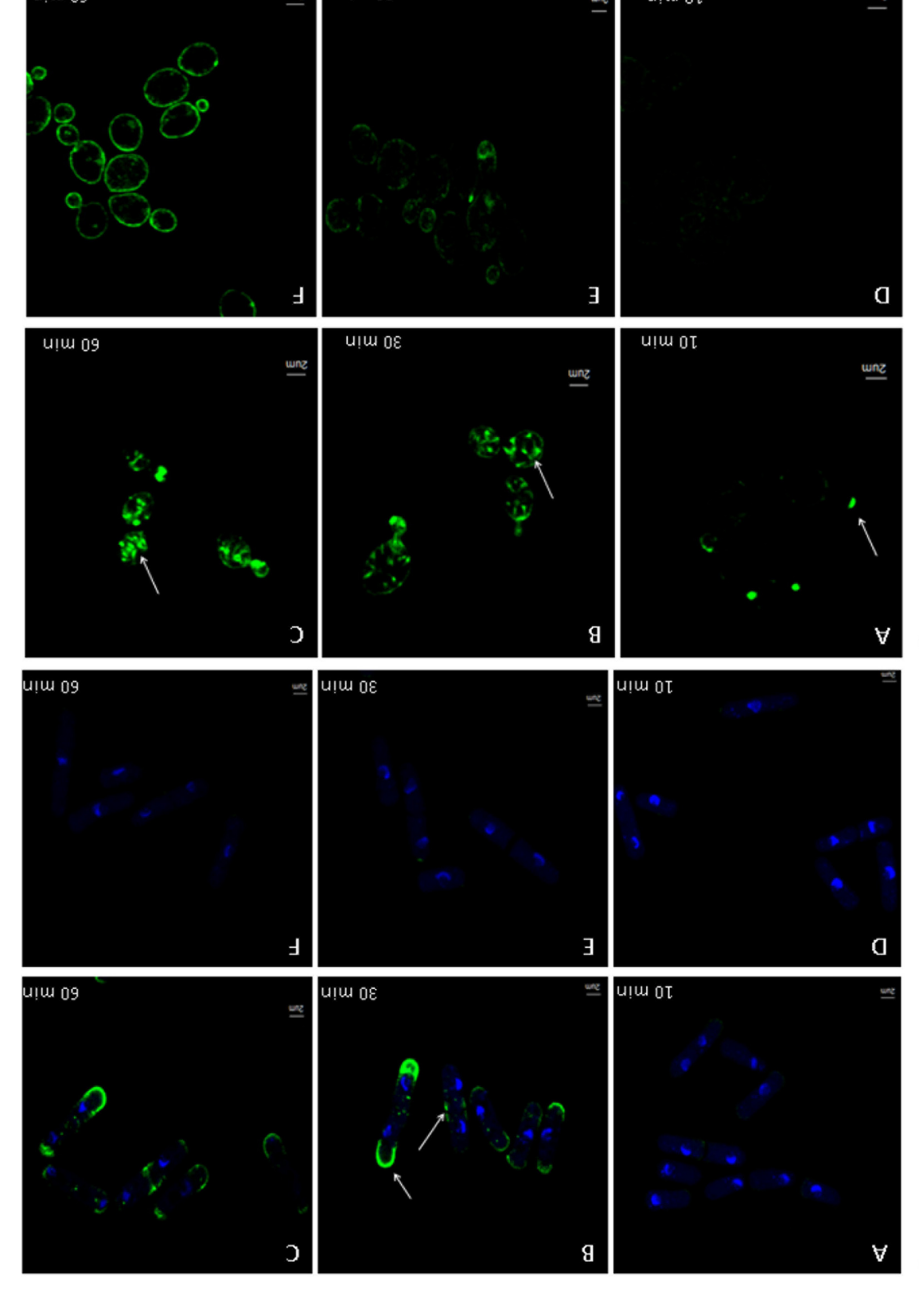
Competition assay of native occidiofungin and alkyne-OF. Time course analyses of alkyne-OF distribution (A to C) and the distribution of alkyne-OF with the competition of native occidiofungin (D to F) in Schizosaccharomyces pombe (a) and Saccharomyces cerevisiae (b). Arrows indicate specific localization patterns of alkyne-OF observed in each cell at 10, 30, and 60 min. When pretreated with native occidiofungin, alkyne-OF does not bind or is restricted to the cellular envelope in Schizosaccharomyces pombe and Saccharomyces cerevisiae, respectively.

*In vivo* visualization of the localization of occidiofungin was performed in intact yeast cells. Time course analysis of S. pombe following alkyne-OF treatment and derivatization with azide Alexa Fluor 488 showed a specific pattern of localization (Fig. 2a). Alkyne-OF produced a faint pattern of staining at the polar tips at 10 min posttreatment, which subsequently increased in intensity at 30 min posttreatment. Strong fluorescence was observed at the polar ends of the cells and at the septa of dividing cells. A similar assay conducted using S. cerevisiae showed localization of alkyne-OF at the bud tips at the early time points and staining throughout the parent cell at later time points (Fig. 2b). The unique pattern formed was observed to be a combination of striated and inclusion-like structures. In both yeast systems, when cells were pretreated with native occidiofungin prior to the treatment with alkyne-OF, the observed cellular localization patterns disappeared (Fig. 2a and b, panels D, E, and F) indicating that alkyne-OF and occidiofungin compete for the same cellular target. The vesicular pattern observed at the later time points of exposure is indicative of endocytic vesicles coated with actin being circulated through the cell (26, 27). Actin patches in the cells of S. pombe were seen at the cell tips in growing cells and at the division septa in dividing cells. Actin patches recruited to the division septum interact with myosin to form the actomyosin ring, which is instrumental in cell division (28). The time course analyses in both types of fungal cells show the localization of occidiofungin to the regions where actin is known to be localized.

### Perturbation of actin-based functions following occidiofungin exposure.

**FIG 3:**
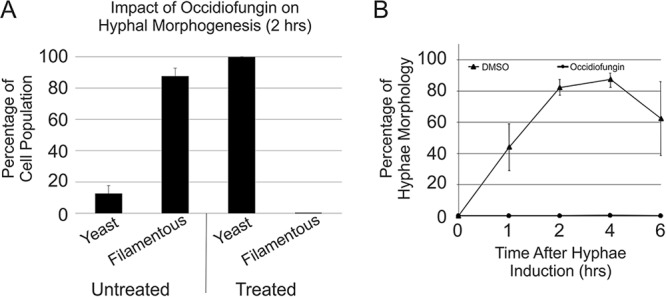
Candida albicans morphology under hypha-inducing conditions. (A) The percentages of cells with a morphology scored as “yeast” or “filamentous” at 2 h. The data are the averages with the standard deviations from >200 cells per sample (*n* = 3) for each treatment condition. (B) The percentages of the cell population that had a hyphal morphology following exposure to DMSO and occidiofungin after 0, 1, 2, 4, and 6 h at 37°C in Spider medium. The data are the averages with the standard deviations from >200 cells per sample (*n* = 2 or 3) for each time point and treatment condition.

**FIG 4:**
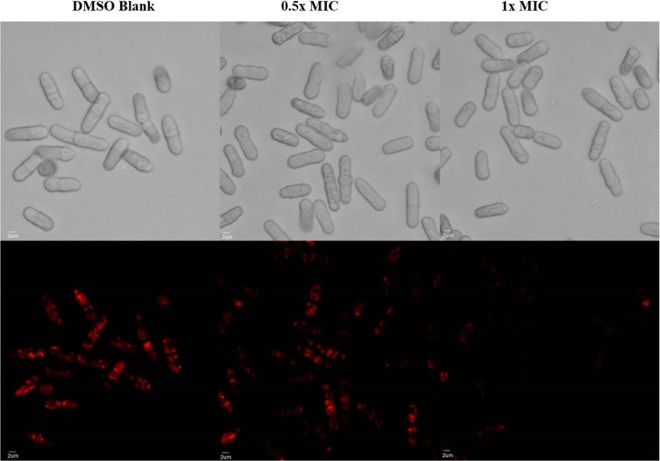
Effect of the native occidiofungin on endocytosis in fission yeast. Differential inference contrast (DIC; top) and fluorescence (bottom) images of cells stained with FM-464 following treatment with the sample blank, 0.5× MIC of occidiofungin, and 1× MIC occidiofungin. FM-464 dye uptake by endocytosis was decreased in a dose-dependent fashion in cells exposed to occidiofungin.

The addition of occidiofungin had no effect on the polymerization or depolymerization properties of F-actin (see Fig. S3). Therefore, additional studies related to the perturbation of actin-based functions were performed to confirm that actin was the biological target of occidiofungin. As a dimorphic fungus, C. albicans can grow as a yeast or hypha, and the ability to switch between these forms is linked to the pathogenicity of the organism (29). Several reports have established that actin cables play an important role in the switch between yeast and hyphal forms (30, 31). The impact of the antifungal on the morphological switching of C. albicans cells was tested. The incubation of C. albicans with a subinhibitory concentration of occidiofungin blocked hyphal formation in cells that were induced to undergo morphological switching (Fig. 3 and Table S5). The morphogenesis of C. albicans from yeast to filamentous forms involves actin dynamics, as treatments with cytochalasin A and latrunculin A or the elimination of myosin I function prevent hyphal formation (32, 33). In C. albicans, the maintenance of the actin scaffold is also necessary for endocytosis, DNA segregation, and cell division (34, 35). To determine whether occidiofungin impacts other cellular activities linked to actin dynamics, we evaluated the effect of occidiofungin on endocytosis in fission yeast by staining cells with FM-464 (Fig. 4). Cells exposed to 0.5× MIC and 1× MIC demonstrated concentration-dependent reductions in stained endocytic vesicles. Actin has also been linked to the proper positioning of the mitotic spindle during cell division, and mutants that lack actin cables exhibit an accumulation of multinucleated cells (36, 37). Within 30 min of exposure, both S. cerevisiae and C. albicans cultures treated with a subinhibitory concentration of occidiofungin had more binucleated cells indicative of a disruption or a delay in nuclear transit through the mother-daughter neck (see Table S3). To further characterize the role of actin in the cellular response to occidiofungin, we analyzed haploid S. cerevisiae mutants deleted for genes linked to actin polymerization and depolymerization. The deletant mutants were chosen for their role in actin organization and dynamics and screened for any deviation in observed activity (increase or decrease in occidiofungin susceptibility). Of the eighteen strains tested, only the Δ*tpm1* mutant showed altered sensitivity to occidiofungin, with the deletion mutant exhibiting a 4-fold resistance to occidiofungin (see Table S4). The observed increase in resistance to occidiofungin in the absence of the *tpm1* gene, which codes for the major isoform of tropomyosin, may be due to the mutant’s increased tolerance of cellular stressors (unpublished data) or a decrease in the cellular growth rate (38). A decrease in cellular growth has previously been linked to occidiofungin resistance (39).

**FIG 5:**
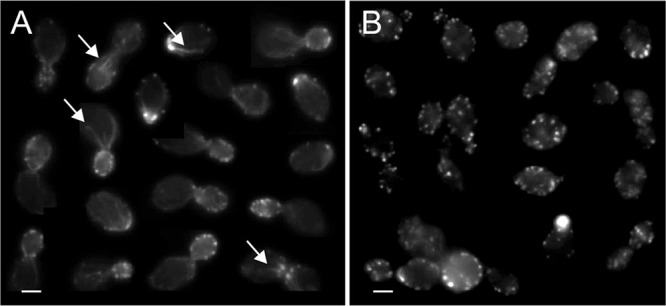
Effects of occidiofungin exposure on the integrity of actin cables in S. cerevisiae cells. Images of cells processed for actin visualization using phalloidin-TRITC from a culture exposed to solvent blank control (DMSO), where actin patches and cables (arrows) are easily identifiable (A), and a culture treated with occidiofungin (0.5× MIC; for 30 min), showing a loss of actin cables and the accumulation of actin aggregates (B). Scale bars, 2 µm.

To directly determine the effect of occidiofungin on actin organization *in vivo*, fluorescence microscopy was carried out on diploid cells of S. cerevisiae exposed to subinhibitory concentrations of occidiofungin. Within 30 min of exposure, an accumulation of actin patches and/or aggregates of F-actin were observed throughout the treated cells with a concomitant loss of actin cables (Fig. 5A and B; see also Table S6). Actin cables are formed by bundling F-actin. In Fig. 5, the punctate structures in these cells are still likely filamentous actin, but occidiofungin disrupts the organization of F-actin to form cables at subinhibitory concentrations.

### *In vitro* analysis of the interaction of occidiofungin with purified actin.

**FIG 6:**
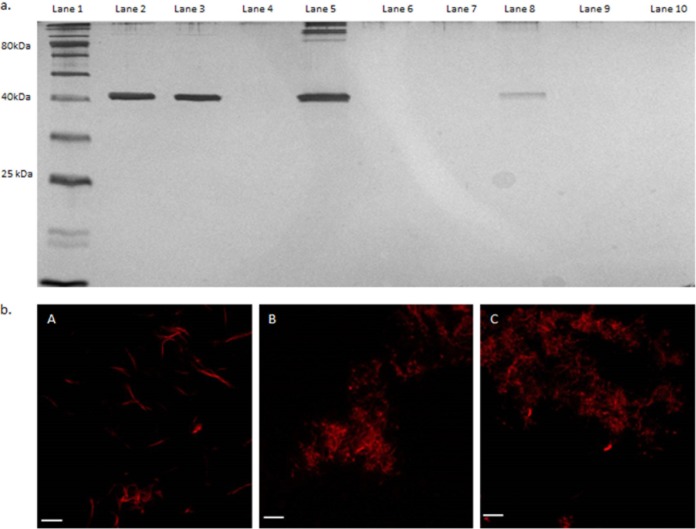
*In vitro* interaction of occidiofungin with F- and G-actin. (a) Affinity pulldown of actin using alkyne-OF. Lane 1, ladder; lane 2, 100 ng pure F-actin; lane 3, 100 ng pure G-actin; lane 4, empty; lane 5, F-actin treated with alkyne-OF; lane 6, F-actin treated with native occidiofungin; lane 7, F-actin treated with DMSO; lane 8, G-actin treated with alkyne-OF; lane 9, G-actin treated with native occidiofungin; lane 10, G-actin treated with DMSO. (b) Fluorescence microscopy of the effect of occidiofungin treatment on actin filaments visualized by fluorescently labeled phalloidin. (A) Actin filaments treated with solvent blank (DMSO). (B) Actin/native occidiofungin (24 μg actin:4 μg native occidiofungin). (C) Actin/native occidiofungin (24 μg actin:8 μg native occidiofungin). Scale bar, 5 µm.

**FIG 7:**
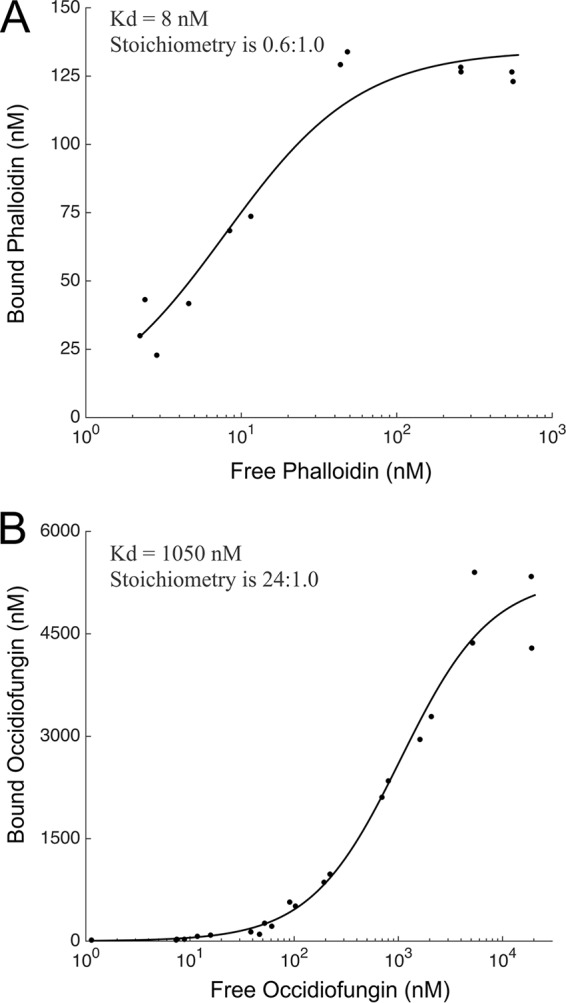
Cosedimentation assay demonstrating the binding of occidiofungin to actin. (A) Binding curve of phalloidin to actin (*K_d_* = 8 nM; the stoichiometry [ligand: protein] is 0.6:1.0). (B) Binding curve of occidiofungin to actin (*K_d_* = 1 μM; the stoichiometry [ligand: protein] is 24:1). The graph is plotted between the amount of free occidiofungin obtained in the supernatant of the cosedimentation assay and the amount of bound occidiofungin obtained from the actin pellet. The data were fit to a standard Langmuir binding isotherm of the form: [X]bound = [X]×S/(*K_d_* + [X]), where S is the maximal X bound, *K_d_* is the dissociation constant, and X is the concentration of free ligand.

The biotinylation of alkyne-OF following incubation with rabbit skeletal muscle F- or G-actin and streptavidin agarose beads was performed to determine whether occidiofungin directly associated with purified actin *in vitro*. F- or G-actin incubated with the wild-type occidiofungin or dimethyl sulfoxide (DMSO) was used as the control for a potential nonspecific interaction of actin with the agarose beads. The eluant from the biotinylated alkyne-OF produced a single band at approximately 42 kDa, the expected size for actin. As shown in Fig. 6a, the biotinylation of alkyne-OF was required for the copurification of F- or G-actin with the streptavidin beads (lanes 5 and 8), as actin was not detected in the control lanes for which actin was exposed to native OF or the carrier solvent DMSO (lanes 6, 7, 9, and 10). In this *in vitro* interaction assay, occidiofungin was shown to directly bind to F- or G-actin. To further support this observation, we performed a cosedimentation assay, which is commonly reported for identifying actin-associated proteins (40, 41). Phalloidin was used as a positive control in the assay (Fig. 7) and had an estimated dissociation constant (*K_d_*) of 8 nM with a saturation of binding ratio of 0.6 phalloidin to 1 actin monomer. These values are corroborated by previous reports (42). The saturation of binding for occidiofungin was 24 molecules of occidiofungin to each actin monomer. Even with the large number of occidiofungin molecules bound to actin, the estimated macroscopic dissociation constant (*K_d_*) value was still 1.0 μM. The higher *K_d_* value is attributed to a 50-fold increase in the amount of occidiofungin bound to actin compared to that of phalloidin. This means that the microscopic dissociation constant (which captures the affinity of one occidiofungin molecule binding to actin) is significantly lower.

Confocal microscopy with the fluorophore Acti-stain 670 phalloidin was carried out to visualize the impact of occidiofungin or alkyne-OF on actin filaments. The microscopic evaluation revealed that F-actin was still present but that the morphology of F-actin changed in the presence of occidiofungin in a dose-dependent manner (Fig. 6b). When an alkyne-OF labeled with an azide-functionalized Alexa Fluor 488 dye was used, the interaction between occidiofungin and F-actin was directly observed (see Fig. S4). Similar to that shown with labeled phalloidin, F-actin also aggregated following exposure to occidiofungin. The fluorescence visualization of this interaction following treatment with alkyne-OF and native occidiofungin demonstrated a high degree of aggregation of the filaments which was not observed in the controls. Therefore, the binding of occidiofungin to F-actin alters its structural morphology and perturbs actin-based functions within the cell, leading to apoptosis.

### Efficacy of occidiofungin in treating a murine model of vulvovaginal candidiasis.

**FIG 8:**
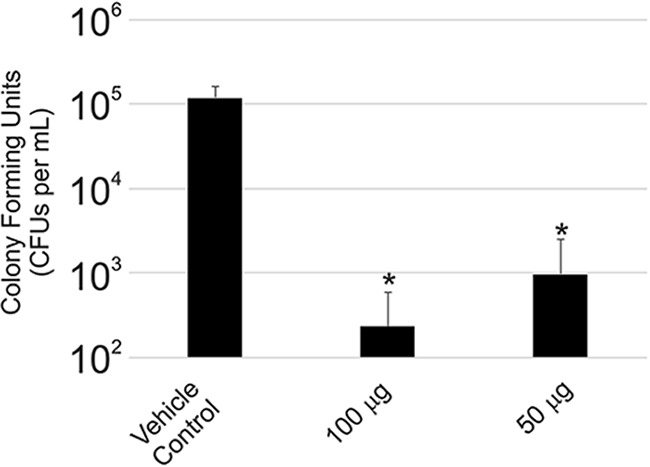
Efficacy of occidiofungin in treating murine vulvovaginal candidiasis. CFUs per ml of Candida albicans in the control group of mice compared to that for the groups treated intravaginally with different concentrations of occidiofungin in 0.3% noble agar. Error bars represent standard deviations.

Six- to eight-week-old BALB/c mice that were intravaginally infected with C. albicans were dosed once per day with occidiofungin for 3 days. The occidiofungin-treated groups were compared to a vehicle control group. Three groups of six mice were intravaginally treated with 100, 50, and 0 µg of occidiofungin suspended in 0.3% Noble agar. The occidiofungin-treated groups showed a 2-log reduction in fungal load (Fig. 8), which was statistically significant from the vehicle control (*P* < 0.001). However, there was no statistically significant difference between the two treated groups (*P* = 0.33), suggesting that the lower limit of occidiofungin dosing was not achieved in the experiment. During the course of the study, the mice were examined for outward signs of distress or irritation. No behavioral changes, including sluggishness, stretching, or reluctance to consume food, were observed. Furthermore, no vaginal bleeding or swelling was observed following treatment.

## DISCUSSION

We have demonstrated that occidiofungin directly interacts with actin, causing a disruption in normal cellular actin-based functions. The *in vivo* data in yeast demonstrate that following treatment, the compound accumulates in areas rich in actin. In addition, the disruption of cables throughout the fungal cells following exposure to subinhibitory concentrations of occidiofungin can be visualized. This interaction with actin can be observed *in vitro* via pulldown assays and cosedimentation studies. These studies establish the direct interaction of occidiofungin with actin with a dissociation constant similar to those of other well-established actin binding proteins, such as α-actinin, tropomyosin isoforms, and fimbrin (43–45). From the binding studies and *in vitro* microscopy assays, it is evident that occidiofungin likely assembles into a large complex around F-actin. The formation of self-assembled complexes was previously observed with several lipopeptide antibiotics (46). Additionally, cellular processes that rely on the maintenance of the actin cytoskeleton, such as endocytosis, hyphal formation, and nuclear DNA positioning, were shown to be disrupted with the addition of occidiofungin. We also demonstrated that occidiofungin is capable of treating a murine vulvovaginal candidiasis infection without any signs of toxicity. Furthermore, occidiofungin demonstrated efficacy at a concentration that is 10-fold lower than that of azole-based treatment methods (47).

We hypothesize that occidiofungin binding to F-actin interferes with the binding of other actin-associated proteins, leading to the disruption of cellular activities involving actin dynamics. The determined binding affinity of occidiofungin is consistent with the low micromolar inhibitory activity reported in Table S1 in the supplemental material. Actin is estimated to be at a concentration >50 µM in yeast, and so the submicromolar binding affinity of occidiofungin means that the vast majority of occidiofungin that enters the cytosol will bind to actin. Furthermore, this suggests that the number of bound occidiofungin molecules to actin that contributes to its antifungal activity is at subsaturation. Cables are necessary for a multitude of cellular functions, including hyphal formation (disrupted following treatment, as seen in Fig. 3), endocytosis (reduced following treatment, as seen in Fig. 4), and the proper positioning of the mitotic spindle during cell division (accumulation of multinucleated cells following treatment, as seen in Table S3 ). The lack of an observed effect on occidiofungin activity with the deletion of the vast majority of the major actin-associated proteins (Table S4) suggests that they are not directly involved in the observed inhibitory activity of occidiofungin. Studies aimed at understanding the events following the binding of occidiofungin to actin will need to be conducted to determine the exact connection to the induction of apoptosis. Furthermore, the localization pattern observed in the microscopy studies performed with S. cerevisiae and S. pombe demonstrates the specificity of occidiofungin to F-actin. The cosedimentation assays and the localization studies indicate that the primary target of occidiofungin in the yeast cell is actin.

Recent studies have shown that the dynamic nature of actin is necessary to maintain the cellular functions in which actin is involved, such as endocytosis, mitochondrial transport, and growth (35). Previous reports suggest that the stabilization and aggregation of actin leads to the induction of a Ras-cAMP-protein kinase A (PKA) pathway which causes mitochondrial destabilization and the production of ROS (31, 48, 49). We demonstrated that occidiofungin directly interacts with actin. The loss of actin cables, as observed following occidiofungin treatment, may trigger a cascade of events leading to the release of reactive oxygen species. The release of ROS is a known precursor for the onset of apoptosis in yeast (50). A newly formed bud contains a large pool of actin, which coordinates the retrograde transport of vesicles along the actin cable into the mother cell (35). Microscopy studies and assays on actin-associated cellular processes support the hypothesis that occidiofungin binding disrupts the normal cellular function of actin. Furthermore, bud tips in S. cerevisiae are rich in actin patches which are necessary to carry out cellular functions, such as cell division and endocytosis (35). Occidiofungin was observed to localize to these cellular areas in the *in vivo* microscopy experiments.

Natural products with *in vitro* antifungal properties that target the actin cytoskeleton were previously reported (51). To date, the few actin binding antifungal compounds, including jasplakinolide (52, 53) and halichondramide (54–56), are limited by their toxicity in animals at doses that would be required to demonstrate an efficacious effect. Occidiofungin, on the other hand, is well tolerated at 5 mg/kg or 20 mg/kg when administered intravenously or subcutaneously, respectively (22, 25). This dose is much higher than the MIC of occidiofungin against several fungal pathogens. Furthermore, blood cells and blood chemistry following the administration of occidiofungin show no serious signs of toxicity (25). It is believed that the growth rates of the cells are attributed to the differences in killing activities. This was later demonstrated with S. cerevisiae, C. albicans, and C. glabrata strains of yeast grown under nutrient-depleted conditions (39). This is also likely why several cancer cell lines were approximately 10-fold more sensitive to occidiofungin than the human fibroblast cell line used as a control (25). We hypothesize that although the cellular target of occidiofungin is highly conserved, the differences in the uptake of occidiofungin into the cell are responsible for the reduced toxicity in animal systems. It is possible that cells that are rapidly dividing, such as the *Candida* spp. in the vaginal cavity and the cancerous cell lines tested, take up occidiofungin much more efficiently than the host cells, leading to a comparably lower toxicity. Future studies will be aimed at understanding the mechanism of uptake and the differences in the effects of the compound.

The low toxicity of occidiofungin combined with its wide spectrum of activities and demonstrable *in vivo* efficacy in treating a VVC fungal infection is unprecedented for any actin binding drug. This is likely due to important differences in the mechanisms behind the modulation of actin dynamics and function. The demonstration of the effect of the compound in eliminating a common fungal infection *in vivo* supports the belief that occidiofungin may constitute a new class of clinically relevant antifungal or anticancer drugs. A better understanding of its entry and the events that lead to apoptosis following the binding of actin may lead to other potentially novel therapeutics.

## MATERIALS AND METHODS

### Characterization of the bioactivity of occidiofungin and alkyne analog.

Occidiofungin was purified from a liquid culture of Burkholderia contaminans MS14 as previously described (21). MIC susceptibility testing was performed according to the CLSI M27-A3 and M38-A2 standards for the susceptibility testing of yeasts and filamentous fungi, respectively. S. cerevisiae deletion mutants were obtained from the commercially available BY4741 deletion library (Thermo Scientific).

The addition of an alkyne reactive group to the primary amine on occidiofungin was performed initially at the Texas A&M Natural Products LINCHPIN Laboratory at Texas A&M University and subsequently at the CPRIT Synthesis and Drug-Lead Discovery Laboratory at Baylor University (see Fig. S1 in the supplemental material). A solution of triethylamine and acetonitrile was prepared by dissolving 10 µl of triethylamine in 11.7 ml of acetonitrile. A stock solution of acetonitrile and the reagents was prepared by adding 2,5-dioxopyrrolidin-1-yl hex-5-ynoate (1.40 mg, 6.69 µmole, 8.2 equivalent) to 1.08 ml of the triethylamine and acetonitrile solution prepared above. Occidiofungin (1 mg, 0.82 µmole, 1 equiv.) was added to the stock solution of acetonitrile and reagents (400 µl) containing triethylamine (0.34 µl, 2.46 µmole, 3 equiv.) and 2,5-dioxopyrrolidin-1-yl hex-5-ynoate (51.6 µg, 2.46 µmole, 3 eq). Occidiofungin was added while stirring, with water (400 µl) added to the mixture as a cosolvent. The resultant mixture was stirred for 3 days at 22°C. Purification of alkyne-OF was performed by high-pressure liquid chromatography (HPLC; gradient, 5%; acetonitrile/water to 95% over 20 min) using a Phenomenex Gemini 5 µm C_18_ 110 A (100 mm by 21.2 mm) reversed-phase column. HPLC analysis of the reaction mixture indicated the complete consumption of the starting material and a new peak in the chromatogram (OF retention time [RT] = 13.6 min; alkyne-OF RT = 15.0 min). The structure of the isolated product was confirmed by mass spectrometry (MS) and nuclear magnetic resonance (NMR) analyses (see Fig. S5). A structural analysis of the derivatized product revealed that occidiofungin (OF-B) and burkholdine (Bk-1215) isolated by Lin et al. (57), are likely identical products. The activity of the purified alkyne-OF against Saccharomyces cerevisiae BY4741 and Schizosaccharomyces pombe 972h- was compared to that of the native compound by using the CLSI M27-A3 method. Additionally, the activity of the alkyne derivatized occidiofungin was tested against a higher density (optical density at 600 nm [OD_600_] of 0.6 to 0.8) of cells for both yeasts. Assays for terminal deoxynucleotidyltransferase-mediated dUTP-biotin nick end labeling (TUNEL) (APO-BrdU TUNEL assay kit; Life Technologies), phosphatidylserine externalization (Annexin-V-Fluos staining kit; Roche), and ROS detection (dihydrorhodamine 123; Sigma) were carried out as previously described (24).

### Actin-associated bioactivity assays.

The hyphal induction assay was performed as previously reported with minor modifications (58). Candida albicans strain ATCC 66027 was grown in yeast extract-peptone-dextrose (YPD) medium at 30°C for 48 h to reach saturation with an OD_600_ of 17 to 19. Cells were diluted into fresh Spider medium (1% nutrient broth, 1% mannitol, 0.2% K_2_HPO_4_) to obtain an OD_600_ of 0.05 (∼0.5 × 10^6^ to 1 × 10^6^ cells/ml) and incubated at 37°C with shaking to induce hyphal formation. Immediately prior to the 37°C incubation, occidiofungin (1 µg/ml; 0.5× MIC) or an equal volume of DMSO was added to the cultures. Aliquots were removed at 0-, 1-, 2-, 4-, and 6-h time points and fixed in 3.7% formaldehyde for later analysis of cell morphology by light microscopy. More than 200 cells were examined for each time point, and cells were scored as having either a yeast or filamentous form, with cells showing any outgrowth considered filamentous. DNA segregation assays were performed using a mid-log culture of S. cerevisiae (BY4743, diploid) and C. albicans (ATCC 66027). The cultures were diluted in fresh YPD medium to obtain OD_600_s of 0.095 (∼1 × 10^6^ to 1.5 × 10^6^ cells/ml) and 0.05 (0.5 × 10^6^ to 0.8 × 10^6^ cells/ml), respectively. A blank vehicle control (DMSO) or occidiofungin (1 µg/ml; 0.5× MIC) was added, and the cultures were placed at 30°C with shaking. Samples were removed at 0.5, 1, and 2 h, and the cells fixed by adding formaldehyde (3.7%). The cells were washed in phosphate-buffered saline (PBS) and permeabilized by adding an equivalent volume of PBS containing 0.2% Triton X-100 for 30 min at room temperature. The cells were washed thrice in PBS, added to concanavalin A-treated glass slides, and overlaid with Vectashield plus DAPI (4′,6-diamidino-2-phenylindole). Images were viewed using a Nikon 50i fluorescence microscope with a 100× oil immersion objective and DAPI filter. Cells were scored on the basis of bud morphology and DNA location. One-way analyses of variance (ANOVAs) with *post hoc* Tukey’s honestly significant difference (HSD) testing were used to determine statistically significant differences in the distributions between treated and untreated cells. In addition, FM-464 uptake assays were used to observe endocytosis. Three 1-ml aliquots of S. pombe (OD_600_ of 0.6 to 0.8) were treated with 1 μl DMSO, 0.5 μg/ml (0.5× MIC), or 1 μg/ml (1× MIC) of native occidiofungin for 30 min at 30°C. The cells were washed thrice with PBS and resuspended in YPD medium containing 8 mM FM-464 (Thermo Fisher Scientific). The cells were incubated for 60 min at 30°C, followed by two washes in PBS before visualizing on an Olympus FV1000 confocal microscope with a 40×/0.9 dry objective. Lastly, actin polymerization and depolymerization assays were performed using the Actin polymerization biochem kit (catalog number BK003; Cytoskeleton, Inc.) according to the manufacturer’s instructions.

### *In vivo* and *in vitro* actin binding studies.

S. pombe and S. cerevisiae cells (OD_600_ of 0.6 to 0.8) were incubated with 1× MIC of alkyne-OF at 30°C, with samples removed at 10, 30, and 60 min postincubation. The cells were washed in PBS and fixed with 3.7% formaldehyde. The cells were permeabilized by adding 0.5% Triton X-100. The cells were washed with PBS before the Click reaction with azide-derivatized Alexa Fluor 488 (Click-iT EdU imaging kit; Thermo Fisher Scientific). The cells were visualized with an Olympus FV1000 confocal microscope. A competition assay was carried out by pretreating cells with 1× MIC of the native occidiofungin followed by treatment with alkyne-OF. A mid-log culture of S. cerevisiae (diploid, BY4743) was diluted in fresh YPD medium to an OD_600_ of 0.095 (∼1 × 10^6^ to 1.5 × 10^6^ cells/ml) and occidiofungin (1 µg/ml; 0.5× MIC) or an equivalent volume of DMSO was added. A 6.6 µM concentration of tetramethyl rhodamine isothiocyanate (TRITC)-tagged phalloidin was added to formaldehyde-fixed cells (permeabilized with 0.2% Triton X-100) for 30 min at room temperature. The cells were washed in PBS, added to a concanavalin A-treated glass slides, and overlaid with Vectashield plus DAPI. Images were viewed using a Nikon 50i fluorescence microscope with a 100× oil immersion objective and Texas Red and DAPI filter sets. Random images were captured using a Retiga EXi black and white charge-coupled-device (CCD) camera and Image Q software. All images were captured using the same exposure settings, with the image contrast altered postcapture using CorelDraw.

Purified rabbit skeletal muscle filamentous actin (catalog number AKF99) and G-actin (catalog number AKL95) were purchased from Cytoskeleton, Inc. Actin was reconstituted in Milli-Q water to achieve a stock concentration of 0.4 mg/ml in a buffer that consisted of 5 mM Tris-HCl (pH 8.0), 0.2 mM CaCl_2_, 0.2 mM ATP, 2 mM MgCl_2_, and 5% (wt/vol) sucrose as directed by the supplier. This solution was stored at −80°C in 50-μl aliquots until use. Immediately before use, each aliquot was thawed by placing it in a 37°C water bath for 5 min and then at room temperature. For all studies, 24 μg of F- or G-actin was used with 8 μg alkyne-OF. F-actin filaments were reacted with native occidiofungin for 15 min at room temperature at molar ratios of 1:10 (24 μg actin to 8 μg native occidiofungin) and 1:5 (24 μg actin to 4 μg native occidiofungin). As per the manufacturer’s instructions, Acti-stain 670 phalloidin (Cytoskeleton, Inc.) was used as a control in an *in vitro* actin filament binding study. Actin filaments treated with DMSO (solvent blank negative control) were stained and observed for comparison. The actin filaments were visualized on an Olympus FV1000 confocal microscope. Pulldown assays were performed using alkyne-OF with azide-biotin (Click-iT protein reaction buffer kit; Thermo Fisher Scientific), according to the manufacturer’s instructions. The proteins captured by biotinylated alkyne-OF on streptavidin beads were eluted by boiling in 50 µl of SDS sample loading buffer. The sample was electrophoresed through a 12% SDS gel, and protein bands were visualized by silver staining (Pierce Silver stain kit; Thermo Fisher Scientific) according to the manufacturer’s protocol. F- and G-actin treated with DMSO and native occidiofungin were used as the controls.

The reaction buffer for F-actin (200 nM) and occidiofungin in the cosedimentation study consisted of 5 mM Tris-HCl (pH 8.0), 0.2 mM ATP, and 2 mM MgCl_2_. Occidiofungin was added to the reaction buffer at concentrations ranging from 25 to 25,600 nM. Phalloidin was added to the reaction at concentrations ranging from 25 to 800 nM. The samples were incubated at room temperature for 30 min in thick-wall polycarbonate tubes (349622; Beckman Coulter, CA). After the incubation, the samples were centrifuged at 100,000 rpm at 4°C for 20 min to pellet F-actin and bound occidiofungin (Beckman TL-100 ultracentrifuge). Both the supernatant and the pellet were extracted with two sample volumes of solvent consisting of a combination of 70% acetonitrile (ACN) containing 0.1% trifluoroacetic acid (TFA) and 30% methanol containing 0.4% formic acid. The extracted samples were freeze-dried and brought up to 100 μl with 50% ACN with 0.1%TFA containing a 1-μg/ml concentration of the internal standard. A TSQ Quantum access triple quadrupole mass spectrometer was used to quantify the amount of occidiofungin within each sample. An analog of occidiofungin served as the internal standard for native occidiofungin, while native occidiofungin served as the internal standard for phalloidin.

### Efficacy studies using occidiofungin.

The murine model of vulvovaginal candidiasis has been reported (59). A variation of this method was used. Three groups of six mice were used to evaluate two concentrations of occidiofungin (100 μg and 50 μg) and vehicle control (0.3% Noble agar). Briefly, 6- to 8-week-old BALB/c mice were treated subcutaneously with 200 ng per mouse of β-estradiol 17-valerate 3 days prior to inoculation with C. albicans (day −3). A subcutaneous dose of estradiol was administered every 3 days (day 0 [D0] and D3) until the end of the experiment to induce pseudoestrus. Intravaginal inoculations of approximately 20 μl of 2.5 × 10^6^ CFU/ml of C. albicans were performed on D0 of the VVC study. On the same day of inoculation (D0), another subcutaneous injection of estradiol was made. A lyophilized powder of occidiofungin containing either 100 μg or 50 μg of occidiofungin was suspended in 20 μl of warm 0.3% Noble agar before the intravaginal inoculations. Drug treatments were performed on D2, D3, and D4 of the study. On day 5 (D5), the vaginal lumen was lavaged with 100 μl of sterile PBS with a 200-µl pipette tip. Serial dilutions and total CFU per vaginal lavage were determined by plating on YPD plates containing 50 μg/ml of chloramphenicol. Body weights, signs of vaginal irritation such as swelling or bleeding, and clinical signs of discomfort (stereotypical stretching behavior) were monitored. Statistical analyses (*t* tests) were performed to compare the control group to the treated groups and to compare differences between treated groups. All the analyses were 2 sided, with a *P* value of <0.05 considered statistically significant.

### Ethics statement.

Research Compliance’s Animal Welfare Office (AWO) supports Texas A&M's Institutional Animal Care and Use Committees (IACUC), through which all faculty, staff, and students using animals, regardless of location or funding, must obtain approval before activities begin. The committee approved the study titled Determination of efficacy of occidiofungin in the treatment of vulvovaginal candidiasis (IACUC 2017-0164). The specific national guidelines followed by Texas A&M’s AAALAC-accredited animal facilities are the USDA animal welfare assurance regulations (Texas A&M registration number 74-R0012) and PHS NIH Guidelines (Texas A&M registration number A3893-01).

## Supplementary Material

Supplemental file 1
