# Supplementary material for: A Novel Actin Binding Drug with In Vivo Efficacy
Source: Antimicrob Agents Chemother. 2018 Dec 21;63(1):e01585-18. doi: 10.1128/AAC.01585-18 (PMC6325233; doi:10.1128/AAC.01585-18)
Supplement: Supplemental file 1 [file 193d6511dd620c789682b8a0222b0941_AAC.01585-18-s0001.pdf]

Supplementary Material

Supplementary Tables

S1 Table: Activity of occidiofungin against filamentous and non-filamentous fungi.

S2 Table: Activity of alkyne-OF compared to native occidiofungin

S3 Table: Occidiofungin exposure results in nuclear segregation defects in mitotic cultures of *S. cerevisiae* and *C. albicans*. Nuclear DNA was scored by DAPI staining of fixed cells treated with 0.5X MIC occidiofungin for 0.5, 1, and 2 hours at 30˚C. Cells were binned into one of four categories based on bud morphology and DNA localization and the percentage of cells in each category are reported. Approximately 200 cells were scored for each time point and data from two separate experiments are shown. Statistically significant differences are given for each category compared to the corresponding untreated samples (*; p-value < 0.05, **; p-value < 0.01).

S4 Table: Activity of occidiofungin against *S. cerevisiae* mutants deleted for genes linked to actin polymerization and depolymerization.

S5 Table: Occidiofungin exposure inhibits morphology switching in *C. albicans*. Cells from a 48hr-saturated culture were diluted into Spider media containing DMSO (Untreated) or occidiofungin (1µg/ml; 0.5X MIC) and placed at 37˚C to induce hyphae formation. Cell morphology was scored as either yeast (Y) or hyphae (H) by microscopy of fixed cells at 0, 1, 2, 4, and 6 hours post switching. The cell counts from 2-3 biological replicates are shown. The percent of yeast and hyphal cells in the population was calculated and given in %Y/H column.

S6 Table: Cells exposed to occidiofungin lack actin cables. The data is presented for cells treated with 0.5X MIC occidiofungin (1µg/ml) for 30 and 60 minutes. *S. cerevisiae* cells were scored as either having or not having detectable actin cables following fluorescence microscopy using TRITC-labeled phalloidin. Cell number, average, and standard deviation are presented for two independent replicates.

Supplementary Figures

S1 Figure: Scheme of chemical addition of alkyne group to occidiofungin B and mass determination of alkyne-OF B.

S2 Figure: Induction of apoptosis by alkyne-OF: The 'DMSO' and 'H_2O_2' columns represent the negative and positive controls, respectively. The 'Native OF' column corresponds to cells treated with 1x MIC quantity of native occidiofungin and the last two panels represent treatment of cells with alkyne-OF at the concentration indicated. A) Externalization of phosphatidylserine demonstrated by the fluorescence of Annexin-V-Fluorescein, B) Release of reactive oxygen

species indicated by the formation of rhodamine from dihydrorhodamine 123 and C) Double stranded breaks visualized by TUNEL assay, following treatment with native and alkyne-OF.

S3 Figure: Effect of occidiofungin on actin (a) polymerization and (b) depolymerization *in vitro*. Symbols are as follows: ♦- G-buffer (control), ■ - G-buffer and pyrene actin, ▲ - Test buffer (1.5% β-cyclodextrin in PBS) and pyrene actin (control), X - 20 μL of test buffer containing 20 μg of occidiofungin and pyrene actin.

S4 Figure: Visualization of actin filaments: a) Untreated F-actin filaments stained with phalloidin 670 dye; Alkyne-OF treated F-actin filaments stained with azide derivatized AlexaFluor488 [(b)- (40x); (c)- (100x)]

S5 Figure: Carbon and proton assignments of alkyne subunit. Complete carbon and proton assignments were made using the NMR data shown above and HSQC NMR. All data are consistent with the structure indicated.

S1 Table: Activity of occidiofungin against filamentous and non-filamentous fungi.

Species	Occidiofungin (µg/mL)								Voriconazole	Fluconazole
	24 hours		48 hours		72 hours		96 hours		MIC (µg/mL)	MIC (µg/mL)
	50%	100%	50%	100%	50%	100%	80%	100%		
**Trichophyton mentagrophytes* 10207							1	2	0.25	>16
Trichophyton mentagrophytes 28556							1	2	0.06	>16
Trichophyton mentagrophytes 28641							1	2	0.06	16
[&]*Trichophyton rubrum* 11199							1	2	0.008	0.25
Trichophyton rubrum 28658							1	2	0.03	2
Trichophyton rubrum 28659							1	2	0.03	2
Rhizopus microsporus 28506	4	8	-	8					16	
Rhizopus oryzae 28403	4	8	-	8					>16	
Rhizopus microsporus 27785	2	4	-	8					>16	
Mucor circinelloides 19445	4	8	4	8					>16	
Mucor racemosus 27784	2	4	-	4					>16	
Mucor fragilis 27782	2	4	-	4					>16	
Fusarium solani 28386	2	4	-	4					>16	
Fusarium oxysporum 27718	2	4	-	4					>16	
Fusarium solani 18749	2	4	2	4					>16	

Aspergillus flavus 28517	-	4	-	4					1	
Aspergillus flavus 28455	2	4	-	4					2	
Aspergillus flavus 28445	2	4	-	4					2	
Aspergillus fumigatus 28434	-	4	-	4					1	
Aspergillus fumigatus 28435	-	2	-	2					1	
Aspergillus fumigatus 28436	2	4	2	4					1	
[#]*Candida albicans* 23512	-	1	-	2						32
Candida albicans 28200	4	8	4	8						8
Candida albicans 28102	-	2	-	2						0.125
[#]*Candida glabrata* 27243	2	4	-	4						64
Candida glabrata 25742	-	2	-	2						4
Candida glabrata 28271	4	8	4	8						>64
Candida krusei 9541	2	4	-	4						16
[#]*Candida krusei* 28415	4	8	4	8						64
Candida krusei 28570	4	8	4	8						16
[+]*Candida parapsilosis* 2006	2	4	-	4						0.125
Candida parapsilosis 28364	4	8	4	8						0.25
Candida parapsilosis 28174	-	4	-	4						0.25
Candida tropicalis 9624	-	2	-	2						0.25
Candida tropicalis 28272	4	8	4	8						0.125

Candida tropicalis 28478	4	8	4	8						0.125
Candida auris MRL# 35646	0.125	0.25								
Candida auris MRL# 35651	0.125	0.25								
+*Cryptococcus neoformans* 19526					-	2				4
Cryptococcus neoformans 27708					-	2				2
Cryptococcus neoformans 28446					-	1				4

‘50%’, 80%, and ‘100%’ indicates inhibition endpoints. National Committee for Clinical Laboratory Standards (NCCLS) has established different endpoint criteria for MIC determination that correlate with effectiveness of treatment outcome.

‘-‘ indicates absence of isolates with 50% inhibition endpoint.

Shaded regions indicate antifungal resistant strains: ‘*’ indicates itraconazole resistance, ‘&’ indicates terbinafine resistance, ‘#’ indicates fluconazole resistance and ‘+’ indicates caspofungin resistance.

S2 Table: Activity of alkyne-OF compared to native occidiofungin

Strain	MIC (μg/mL)	
	Native occidiofungin	Alkyne-occidiofungin
Saccharomyces cerevisiae BY4741	0.125	1
Schizosaccharomyces pombe 972h-	0.0625	0.5

S3 Table: Occidiofungin exposure results in nuclear segregation defects in mitotic cultures of *S. cerevisiae* and *C. albicans*. Nuclear DNA was scored by DAPI staining of fixed cells treated with 0.5X MIC occidiofungin for 0.5, 1, and 2 hours at 30˚C. Cells were binned into one of four categories based on bud morphology and DNA localization and the percentage of cells in each category are reported. Approximately 200 cells were scored for each time point and data from two separate experiments are shown. Statistically significant differences are given for each category compared to the corresponding untreated samples (*; p-value < 0.05, **; p-value < 0.01).

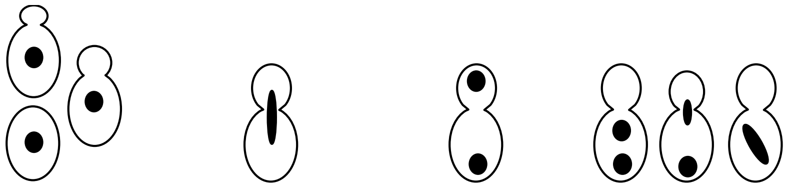

S. cerevisiae

Untreated	80.4 ± 2.6	3.9 ± 1.2	13.7 ± 2.5	1.4 ± 0.5
0.5hr	66.9 ± 6.8	0.2 ± 0.3*	20.5 ± 6.5	12.3 ± 0.6**
1.0hr	59.1 ± 7.7**	0.4 ± 0.1*	25.4 ± 4.7*	15.1 ± 2.9**
2.0hr	63.5 ± 2.2*	0.6 ± 0.8*	22.7 ± 2.3	13.3 ± 0.7**

C. albicans

Untreated	62.1 ± 8.8	10.3 ± 3.8	26.6 ± 5.6	1.0 ± 0.4
0.5hr	58.1 ± 6.6	1 ± 0.7*	35.3 ± 6.8	5.6 ± 0.4
1.0hr	62.9 ± 4.2	0.9 ± 0.1*	28.1 ± 1.3	8.1 ± 2.8*
2.0hr	61.9 ± 7.7	0.4 ± 0.6*	26.3 ±12.1	11.4 ± 3.8**

S4 Table: Activity of occidiofungin against *S. cerevisiae* mutants deleted for genes linked to actin polymerization and depolymerization.

S. cerevisiae strain	Gene function	MIC (µg/ml)
BY4741		0.25
Δ*bni1*	Actin nucleation, Formin	0.25
Δ*bnr1*	Actin nucleation, Formin	0.25
Δ*myo4*	Actin based motor activity, Type V motor	0.25
Δ*myo5*	Actin based motor activity, Type I motor	0.25
Δ*sac6*	Actin bundling, Fimbrin	0.25
Δ*scp1*	Actin cross linker	0.25
Δ*tpm1*	Actin filament binding, Tropomyosin	1.0
Δ*tpm2*	Actin filament binding, Tropomyosin	0.25
Δarc18	ARP2/3 subunit	0.25
Δcap2	Barbed-end actin capping protein; activity prevents polymerization	0.25
Δabp1	ARP2/3 activator; inhibits barbed-end polymerization	0.25
Δtus1	Rho1p GEF	0.25
Δrgl1	Tus1p cofactor; required for Tus1p localization	0.25
Δrdi1	Rho GDP-inhibitor; regulates Rho1	0.25
Δbem2	Rho-GAP for the CWI pathway; synthetically lethal with Δ*tpm1*	0.25
Δrom1	Rho1-GEF	0.25
Δrom2	Rho1/2-GEF	0.25
Δsac7	Rho1-GAP for plasma membrane fluidity signaling	0.25

S5 Table: Occidiofungin exposure inhibits morphology switching in *C. albicans*. Cells from a 48hr-saturated culture were diluted into Spider media containing DMSO (Untreated) or occidiofungin (1µg/ml; 0.5X MIC; Treated) and placed at 37˚C to induce hyphae formation. Cell morphology was scored as either yeast (Y) or hyphae (H) by microscopy of fixed cells at 0, 1, 2, 4, and 6 hours post switching. The cell counts from 2-3 biological replicates are shown. The percent of yeast and hyphal cells in the population was calculated and given in %Y/H column.

Post switch	Biological replicate	Untreated Yeast	Untreated Hyphae	Untreated % Y/H	Treated Yeast	Treated Hyphae	Treated % Y/H
T=0hr	1	973	0	100/0	973	0	100/0
	2	967	0	100/0	701	0	100/0
	3	422	0	100/0	369	0	100/0
T=1hr	1	223	208	51.7/48.3	816	0	100/0
	2	561	211	72.7/27.3	513	0	100/0
	3	230	298	43.6/56.4	300	0	100/0
T=2hr	1	47	154	23.4/76.6	265	0	100/0
	2	98	531	15.6/84.4	481	0	100/0
	3	98	592	14.2/85.8	488	0	100/0
T=4hr	1	17	224	7.1/92.9	211	1	99.5/0.5
	2	156	740	17.4/82.6	1017	0	100/0
	3	136	895	13.2/86.8	937	2	99.8/0.2
T=6hr	2	826	692	52.2/47.8	813	0	100/0
	3	112	419	21.1/78.9	961	0	100/0

S6 Table: Cells exposed to occidiofungin lack actin cables. The data is presented for cells treated with 0.5X MIC occidiofungin (1µg/ml) for 30 and 60 minutes. *S. cerevisiae* cells were scored as either having or not having detectable actin cables following fluorescence microscopy using TRITC-labeled phalloidin. Cell number, average, and standard deviation are presented for two independent replicates.

Treatment	Total	Cells with actin cables				Cells without actin cables			
		Num	%	Avg	SD	Num	%	Avg	SD
Untreated	156	99	63.46	74.13	15.09	57	36.54	25.87	15.09
	487	413	84.8			74	15.2		
0.5X MIC 30 min exposure	152	2	1.32	2.34	1.45	150	98.68	97.66	1.45
	238	8	3.36			230	96.64		
0.5X MIC 60 min exposure	473	0	0	0.18	0.25	473	100	99.82	0.25
	279	1	0.36			278	99.64		

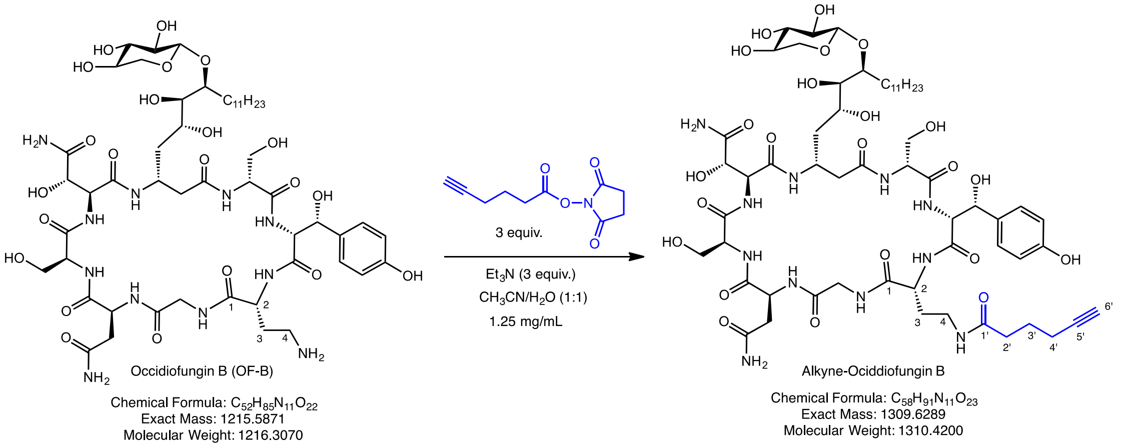

S1 Figure: Scheme of chemical addition of alkyne group to occidiofungin B and mass determination of alkyne-OF B.

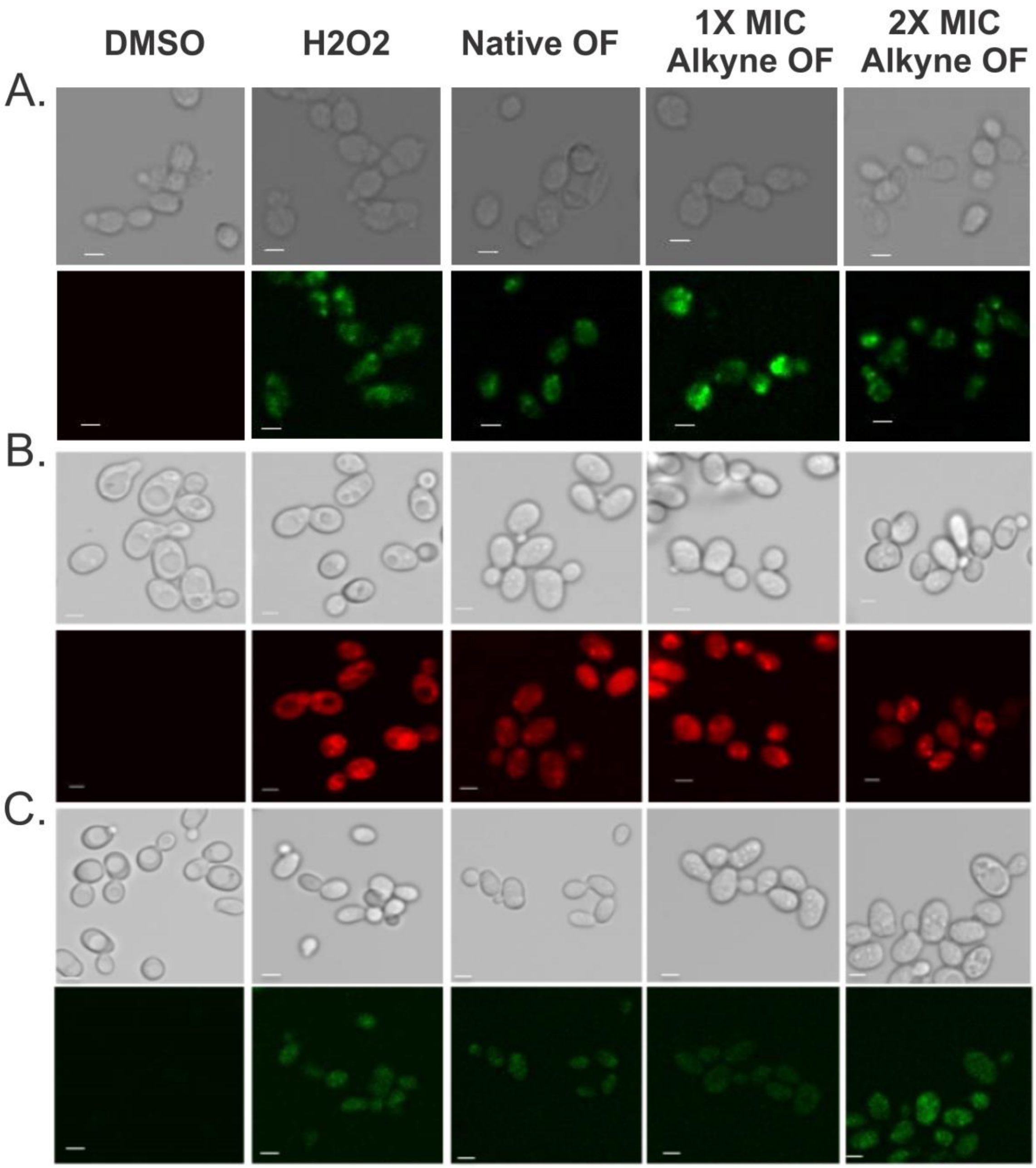

S2 Figure: Induction of apoptosis by alkyne-OF: The 'DMSO' and 'H_2O_2' columns represent the negative and positive controls, respectively. The 'Native OF' column corresponds to cells treated with 1x MIC quantity of native occidiofungin and the last two panels represent treatment of cells with alkyne-OF at the concentration indicated. A) Externalization of phosphatidylserine demonstrated by the fluorescence of Annexin-V-Fluorescein, B) Release of reactive oxygen species indicated by the formation of rhodamine from dihydrorhodamine 123 and C) Double stranded breaks visualized by TUNEL assay, following treatment with native and alkyne-OF.

A.

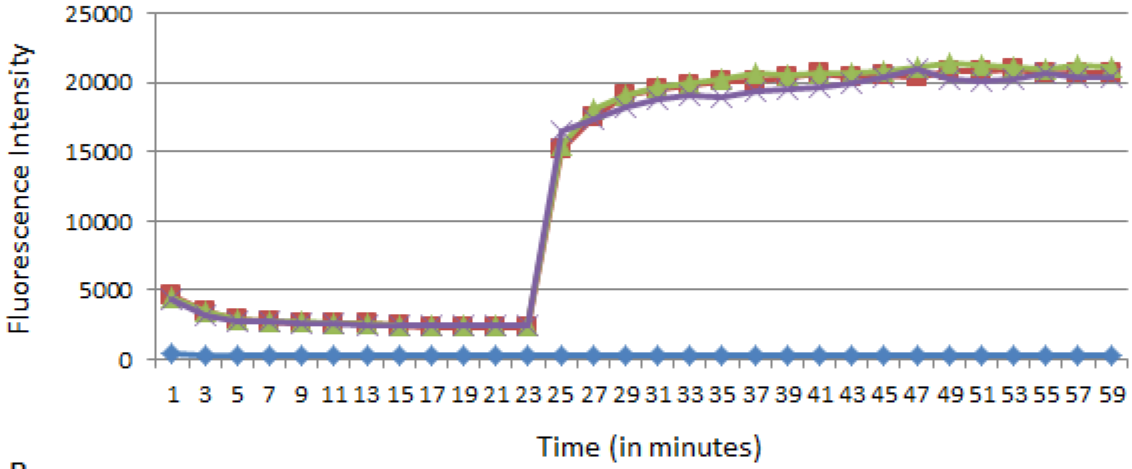

B.

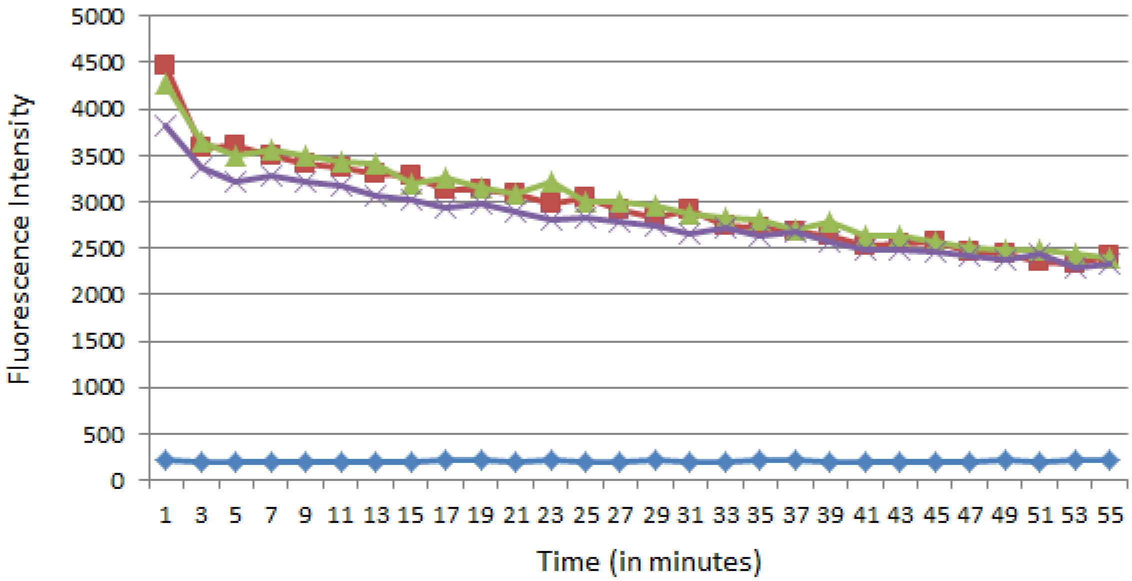

S3 Figure: Effect of occidiofungin on actin (a) polymerization and (b) depolymerization *in vitro*. Symbols are as follows: ♦- G-buffer (control), ■ - G-buffer and pyrene actin, ▲ - Test buffer (1.5% β-cyclodextrin in PBS) and pyrene actin (control), X - 20 µL of test buffer containing 20 µg of occidiofungin and pyrene actin.

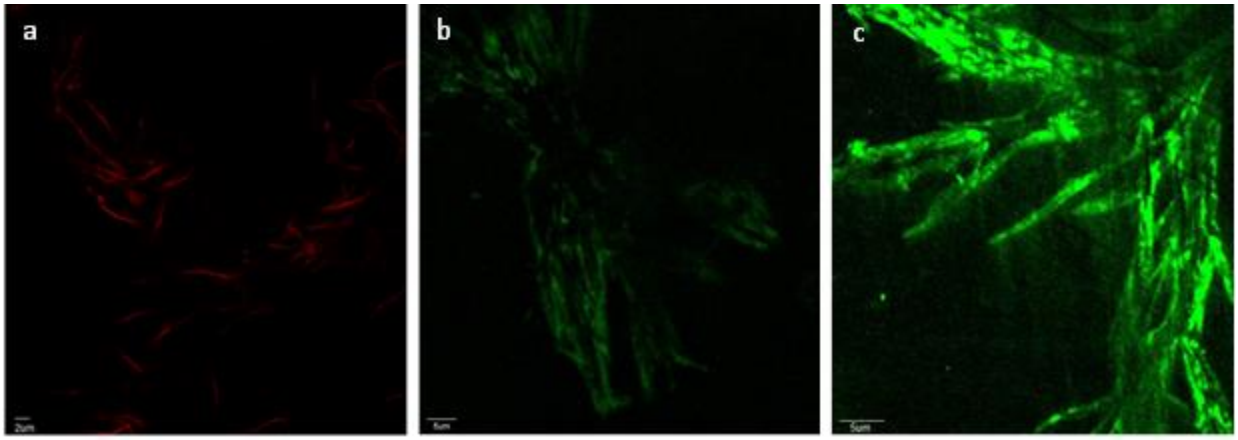

S4 Figure: Visualization of actin filaments: a) Untreated F-actin filaments stained with phalloidin 670 dye; Alkyne-OF treated F-actin filaments stained with azide derivatized AlexaFluor488 [(b)- (40x); (c)- (100x)]

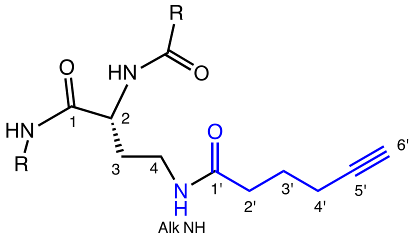

Chemical Shifts in ppm

Position	N-H	1’	2’	3’	4’	5’	6’
^{13}C	-	172.3	34	24.5	17.5	84	71
^{1}H	7.83	-	2.10	1.60	2.10	-	2.70

Position	4	3	2
^{13}C	35.5	31.6	50.8
^{1}H	3.08	1.61, 1.90	4.22

^{13}C assignments from HSQC

S5 Figure: Carbon and proton assignments of alkyne subunit. Complete carbon and proton assignments were made using the NMR data shown above and HSQC NMR. All data are consistent with the structure indicated.
